# Development of a multi-component tobacco cessation training package utilizing multiple approaches of intervention development for health care providers and patients attending non-communicable disease clinics of Punjab, India

**DOI:** 10.3389/fpubh.2022.1053428

**Published:** 2022-12-02

**Authors:** Garima Bhatt, Sonu Goel, Sandeep Grover, Bikash Medhi, Gurmandeep Singh, Sandeep Singh Gill, Leimapokpam Swasticharan, Rana J. Singh

**Affiliations:** ^1^Department of Community Medicine, School of Public Health, Post Graduate Institute of Medical Education and Research (PGIMER), Chandigarh, India; ^2^Public Health Master's Program, School of Medicine and Health Research Institute (HRI), University of Limerick, Limerick, Ireland; ^3^Faculty of Human and Health Sciences, Swansea University, Swansea, United Kingdom; ^4^Department of Psychiatry, Post Graduate Institute of Medical Education and Research (PGIMER), Chandigarh, India; ^5^Department of Pharmacology, Post Graduate Institute of Medical Education and Research (PGIMER), Chandigarh, India; ^6^National Health Mission, Department of Health and Family Welfare Government of Punjab, Chandigarh, India; ^7^Department of Health and Family Welfare, Government of Punjab, Chandigarh, India; ^8^Directorate General of Health Services, Ministry of Health and Family Welfare, Government of India, New Delhi, India; ^9^Department of Tobacco and NCD Control, International Union Against Tuberculosis and Lung Disease (The Union), South-East Asia Office, New Delhi, India

**Keywords:** intervention development, multi-component, tobacco cessation, socio-cultural, behavior, NCD clinics, India

## Abstract

**Background:**

Providing patients with personalized tobacco cessation counseling that is culturally sensitive, and disease-specific from healthcare providers (HCPs) as part of their routine consultations is an approach that could be incorporated, using existing healthcare systems such as the Non-Communicable Disease (NCD) clinics. This paper describes the development of a multi-component culturally tailored, patient-centric, disease-specific tobacco cessation package utilizing multiple approaches of intervention development for healthcare providers and patients attending these clinics in Punjab, India, along with a proposed framework for implementation.

**Methods:**

The proposed intervention package was developed in 6 stages. These included a review of literature for identifying successful cessation interventions for ethnic minority groups, co-production of the package with all stakeholders involved *via* a series of consultative meetings and workshops, understanding contextual factors of the state and ‘factor-in’ these in the package, pre-test of the package among HCPs and tobacco users using in-depth interviews, micro detailing and expansion of the package by drawing on existing theories of the Cascade Model and Trans-Theoretical Model and developing an evolving analysis plan through real-world implementation at two pilot districts by undertaking a randomized controlled trial, assessing implementer's experiences using a mixed-method with a primary focus on qualitative and economic evaluation of intervention package.

**Results:**

A multi-component package consisting of a booklet (for HCPs), disease-specific pamphlets and short text messages (for patients; bilingual), and an implementation framework was developed using the 6-step process. A major finding from the in-depth interviews was the need for a specific capacity-building training program on tobacco cessation. Therefore, using this as an opportunity, we trained the in-service human resource and associated program managers at the state and district-level training workshops. Based on the feedback, training objectives were set and supported with copies of intervention package components. In addition, the role and function of each stakeholder were defined in the proposed framework.

**Conclusion:**

Consideration of tobacco users' socio-cultural and patient-centric approach makes a robust strategy while developing and implementing an intervention providing an enlarged scope to improve care services for diversified socio-cultural communities.

## Background

Non-Communicable Diseases (NCDs) account for a major share of the overall global disease burden claiming 44 million lives annually. NCDs disproportionately affect populations in low- and middle-income nations, which account for 31.4 million global NCD deaths (1). In the Indian sub-continent, NCDs account for 62 percent of all deaths. Besides, the contribution of total “Disability-Adjusted Life Years” (DALYs) from NCDs has increased from 30% (1990) to 55% (2016) (2). In addition, one-eighth of households with NCD burden were pushed to poverty with poverty, deepening the effect to the magnitude of 30.1% among those already below poverty in the year 2017–18 (3).

Article 14 of the WHO- Framework Convention on Tobacco Control envisions member countries take appropriate “demand reduction measures concerning tobacco dependence and cessation” (4). According to Global Adult Tobacco Survey (GATS-2) data, India had the second-lowest quit rate among GATS-2 countries, despite a high prevalence of knowledge about the health consequences of smoking and/or chewing tobacco. Only 55.4 percent of smokers and 50.4 percent of SLT users have ever considered or intended to quit tobacco use (5).

Evidence suggests that a combination of population-wide and individual interventions (pharmacological/non-pharmacological) to modify NCD risk factors are great economic investments because they can prevent the need for more expensive treatment if given to patients early enough (6). Target 3.4 of the Sustainable Development Goals (SDG) aims to reduce premature mortality from NCDs by one-third by 2030. Besides, reducing tobacco use is critical to global efforts to achieve the SDG target (7). The health, social and economic benefits of quitting tobacco use are well established (8). Tobacco cessation is recommended as one of the 'best buys' interventions for preventing and controlling NCDs (9). Evidence from developed countries suggests that brief interventions delivered by diverse health professionals effectively tobacco cessation. Tobacco cessation needs to be urgently expanded by training health professionals in providing routine clinical interventions (10). However, there is a dire need to train healthcare providers to offer brief tobacco cessation interventions (11).

The WHO- Package of Essential Non-communicable Interventions (PEN) also suggests developing programs to address NCD's risk factors, including tobacco in low-resource settings by adapting plans that suit local contexts (including cultural and educational backgrounds) (12). Family members must understand that encouragement from the family can help people adopt healthy living, e.g., cessation of tobacco (6).

Tobacco use is a learned behavior, and nicotine addiction involves biological, behavioral, psychological, and cultural factors. The interplay between these factors results in the continued use of tobacco products among users (13, 14). In an LMIC like India which is culturally diverse and has a dual burden of NCDs and tobacco use, it experiences enormous costs imposed on the nation's health care system. Consequentially, it potentiates stress on the public health care system (15). This calls for low-technology interventions, which could reap future savings in terms of reduced medical costs, improved quality of life, and productivity if delivered effectively. Tobacco's causal association with several long-term conditions [e.g., coronary heart disease (CHD), chronic obstructive pulmonary disease (COPD)] has long been established (16, 17). This requires culture-specific, patient-centric, disease-specific care that reinforces health system strengthening with efficient use of limited health care resources and is sustainable (6).

Data from the Indian Global Health Professionals Students Survey (GHPSS, 2005–2008) between 2005 and 2008 showed a general lack of training among health professionals in patient cessation counseling techniques (18). In a study in Bihar, over two-thirds of medical doctors felt the need to increase their tobacco cessation training (10). Besides, it is essential to address smokeless tobacco cessation in a country like India, where the use of SLT is widespread. Given the higher prevalence of tobacco use in rural populations, extending tobacco cessation services to rural people is imperative. Behavior counseling is applicable and acceptable in rural settings, where access to pharmacotherapy may be limited. In low-resource settings, there is also a need to evaluate cost-effective behavioral interventions, particularly for smokeless forms of tobacco use, for further expansion of tobacco cessation activities (19).

However, there is meager literature on the development or effectiveness of a cessation intervention for NCD patients that is culture-specific, patient-centric, disease-specific, and tested in NCD outpatient settings (20). Therefore, it's crucial to develop that evidence-based interventions are developed and tested in such settings along with co-production with all stakeholders in the development process. This paper describes the development of a multi-component culturally tailored, patient-centric, disease-specific tobacco cessation package utilizing multiple approaches of intervention development for healthcare providers and patients attending non-communicable disease clinics in Punjab, India, and its implementation framework.

## Methods

### Settings

This intervention package was developed for implementation at NCD clinics in Punjab, India. The “NCD clinics” are established by the Government of India under the National Programme for Prevention and Control of Cancers, Diabetes, Cardiovascular Diseases, and Stroke (NPCDCS, 2010–2011) at the district level and Community Health Center Level (CHC). The human resource provisioned at the NCD clinic (at the district hospital) includes a General Physician(1), GNM (2), Technician (1), Physiotherapist (1), Counselor (1), and Data Entry Operator(1). The staff provides emergency and OPD services, counseling, rehabilitative services, and care and management of cancer, diabetes, hypertension, and acute cardiovascular diseases. The clinic shall run on all working days or at least thrice a week (21).

**Figure 1:**
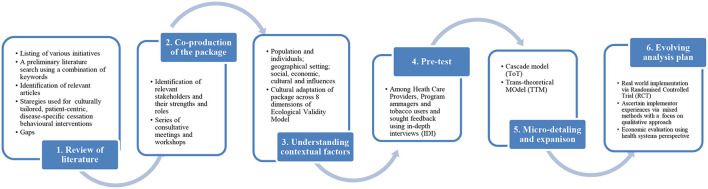
Flow of intervention package development process with major steps.

The proposed culturally-specific, patient-centric, disease-specific intervention package was developed in **6 stages**. These included the following (Figure 1):

1. **Review of literature** for identifying successful cessation interventions for ethnic minority groups.
2. **Co-production** of the package with all stakeholders involved *via* a series of consultative meetings and workshops.
3. **Understanding contextual factors** of the state and ‘factor-in’ these in the package.
4. **Pre-test** of the package among HCPs and tobacco users using in-depth interviews (IDI).
5. **Micro detailing and expansion** of the package by drawing on existing theories of the Cascade Model and TTM model.
6. **Developing an evolving analysis plan** through real-world implementation at two pilot districts by undertaking a randomized controlled trial, assessing implementers' experiences using a mixed methods study, and economic evaluation of the intervention package.

## Stage 1: Review of literature

A preliminary literature search was undertaken using Pubmed, Scopus, and Embase databases by using a combination of keywords like “tobacco cessation,” “tobacco use cessation,” “smoking cessation,” “smokeless tobacco cessation,” “quitting smoking,” “quitting tobacco” “behavior change,” “interventions,” “cessation counseling,” “tailor made,” “culture-specific,” “non-communicable,” “chronic disease,” “disease specific,” “customized,” “behavior therapy,” “behavioral intervention,” “review”. Relevant articles from the preliminary search were identified; related article links were also explored to expand the search till 2017. References from reviews on customized cessation interventions or customized cessation support were also reviewed for the inclusion of relevant studies.

The literature review indicated that most of the culturally-tailored interventions were delivered in the West (22–24) and catered to either one chronic disease or two and primarily among tobacco smokers (25, 26). Besides, the evidence of such an intervention that is tailored to the current disease, and culturally relevant for tobacco users (especially SLT) from Indian settings was limited (20). Literature suggested a need to develop standards for designing culturally tailored interventions besides using theoretical frameworks that can be applied in various healthcare settings. Therefore, research needs to consider such constraints, such as having intervention sites that are accessible to the study participants (27). Several studies used mobile phone text messages for follow-up services and websites to provide culturally and linguistically tailored education (28). Studies also reported the use of bilingual technology platforms to encourage participants to change their lifestyle-related risk factors and demonstrated the importance of involving families to improve efficient care (29). A culturally targeted (CT) smoking cessation intervention conducted among African–American smokers reported high levels of acceptability, better adherence to nicotine replacement therapy, higher quit rates, and better retention and follow-up (30). The update on the Tobacco Use and Dependence Clinical Practice Guidelines (31) stressed the need for additional research to determine the effectiveness of culturally targeted smoking cessation interventions for racial and ethnic minorities (32). In addition, the literature reported that cessation interventions are more effective if they address the different social norms driving tobacco consumption and the difficulties associated with quitting tobacco use. The social context of tobacco users, such as language and culture, can profoundly influence an individual's experience with tobacco, including quitting (33). The existing literature also reported that interventions to improve patient-centered care (PCC) for persons with multimorbidity are in constant growth (34). In a study conducted to elicit patient perspectives on tobacco use treatment in primary care, the participants suggested addressing smoking at every visit and acknowledging and helping patients deal with addiction issues in written and spoken communications (35). Besides, it highlighted that the most important characteristic of PCC is patients' active involvement in the decision-making process (36). Evidence among patients with long-term conditions reported that such a group is generally more receptive to smoking cessation messages with several “teachable moments” during their care (37). Similarly, patients with diabetes, hypertension, and CHD had higher motivation to quit and desire to receive support compared to the general population (38). It further reported that continued smoking with long-term conditions contributes to excess mortality and morbidity by expediting disease progression, worsening outcomes, increasing complication rates, and reducing treatment compliance (39). The Transtheoretical Model (TTM) (40) has been widely used in the development of cessation interventions especially smoking cessation (41) and very few on smokeless tobacco among disease-specific groups, especially in India, where the burden of SLT use remains relatively high (42).

## Gaps in the existing literature

The previous literature from India suggested that there is a need to establish effective tobacco cessation services in diverse health settings with optimal use of existing infrastructure, minimal support, and innovative technology such as mobile phones to improve access. The physicians, in general, lacked knowledge of tobacco cessation protocols and felt uncomfortable or at a loss in their ability to handle the needs of their patients for tobacco cessation (19, 43). Addressing cultural competence through training among the health workforce could help in improving the quality of health service for culturally and ethnically diverse groups (44). However, the evidence does indicate a service provision gap (45). These gaps include a lack of advice to quit from their doctor (46), not realizing the risk of developing diabetic complications with smoking (25), belief that “the damage has already been done” (47), not considering the issue of tobacco use as part of mainstream management (48), etc. There is an urgent need for studies that develop and test well-defined context-specific, tailored, and comprehensive packages for tobacco cessation, especially for high-risk patients (49). Further, the need of the hour is to determine ways to integrate these within existing mechanisms along with the inclusion of economic evaluations. Research is also required to understand the barriers to service provision, as the literature is particularly sparse on the perspectives of healthcare providers (48).

In India, multiple government led and other initiatives including various modules on tobacco cessation under the National Cancer Control Programme (2005) (50), tobacco dependence guidelines (2011) (51), a training manual on tobacco cessation for nurses, health workers and doctors by World Health Organization (52) and a toolkit for delivering five As and five Rs by World Health Organization (53), and various modules under NPCDCS program describing the management of various risk factors including tobacco use (21). However, despite the availability of all these resources for healthcare providers, their preparedness for the delivery of tobacco-cessation services remains low in India (54).

## Stage 2: Co-production of the package

Based on the findings of the literature review, prototypes of package components were developed. The components included a booklet for the health care providers at NCD clinics and disease-specific pamphlets and short text messages for the patients attending these NCD clinics in Punjab. Thereafter, a series of consultative meetings and workshops with stakeholders were carried out and multiple iterations were undertaken.

### Identification of stakeholders and their strengths and roles in the development process

The stakeholders included the following:

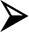
 The program managers of both the programs viz, National Tobacco Control Program (NTCP) and NPCDCS (district and state level),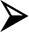 Healthcare providers at the NCD clinics,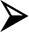 Tobacco users,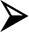 Representatives from civil society,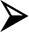 Subject experts of tobacco cessation and NCDs.

Each group of stakeholders was involved at various stages of the development and refinement of the package based on their roles and strengths. In order to ensure sustained support during the development of the intervention package and implementation, decisions were made in collaboration with all. Adaptation decisions were made collaboratively by the original intervention designer, who knew the theory and central operational features of the intervention, and those hosting the new intervention, know their setting, the target population, and the local culture (43). Collaborative working relationships are crucial for making wise decisions regarding fidelity and adaptation (44). The strengths and roles are summarized in the table below (Table 1).

**Table 1:** Strengths and roles of relevant stakeholders.

**Strengths**	**Stakeholder category**	**Roles**
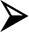 Managerial leadership 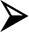 Building partnerships and communication 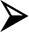 Checking whether the designed package is realistic within the existing contextual settings in which it will operate 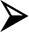 Anticipating and overcoming problems	Program managers	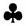 Technical alliance and coalition support for the coproduction of the intervention package 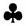 Offering sustained support 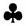 Determining and clarifying what resources are required along with what roles other people can play 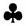 To maximize the use of resources and opportunities. 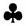 Creating a structure for implementation
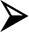 Well aware of characteristics of the population to be catered 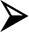 Determining whether the proposed program is realistic and feasible within the context of the organization in which it will be implemented 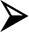 To anticipate and overcome problems	Health care providers	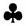 Facilitate refinement and improvements in the package based on real-time context and experience 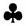 Effectively communicate its success and benefits 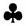 To clarify the resources available 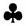 Creating supportive environment 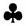 Compatibility or fit with the local setting 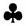 To fit local circumstances 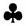 Suggesting capacity-building strategies
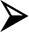 Compatibility or fit with the local setting 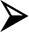 Need of the tobacco users 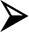 What clicks and appeals to the users and what doesn't 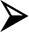 Can share their expectations, perceptions, and beliefs that tobacco use could interact with current medication or NCD condition 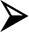 Share their expectations from healthcare providers	Tobacco users	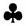 Suggest the modifications required in the intervention package to suit their requirements 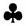 Utilize the information and resources 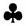 Share their inconvenience, fear, shame, risks, and benefits 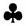 Refine the package for developing targeted messaging 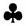 Suggest for patient-centered outcomes 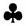 Seek their honest feedback and active participation in discussions
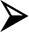 Aware of the characteristics of the population to be reached 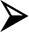 Engaging civil society sectors could contribute to a change in the public perception of an issue	Civil society	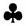 Suggesting regional and contextual anecdotes and case studies illustrating these points 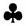 Compatibility or fit with the local setting 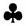 Suggest edits for refinement in the package to fit the cultural relevance
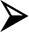 To anticipate and overcome problems. 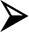 Sound knowledge of theory 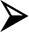 Aware of the gaps 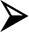 Content and frequency of intervention 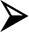 Technical and scientific rigor to the intervention package	Subject experts	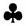 Creating a structure for implementation 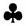 Suggest capacity-building strategies for the 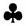 human resource 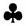 Suggest reviewing, management, and decision-making processes 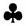 Suggest application of behavior change theories 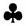 to the package

### Series of consultative meetings and workshops

Workshops expand the perspective to encompass the participants' mutual experience enabling us to undertake an iterative process of design-based research. The workshop as a research approach is an explicit method choice that allows us to iterate, and thus refine and moderate, our research design over time and in different contexts. Consultative meetings with the civil society (*n* = 2), workshop with program managers (*n* = 1), consultative meetings with healthcare providers (*n* = 1), and an advocacy workshop with program managers, administrative authorities, and health café providers (*n* = 1) were undertaken. The objectives of each activity and its outcome are described in Table 2.

**Table 2:** Activities undertaken during consultative meetings and workshops.

**Activity**	**Setting**	**Input**	**Output**
**1**^**st**^ **consultation meeting with CSOs**	**Location:** Department of Community Medicine and School of Public Health, Post Graduate Institute of Medical Education and Research, Chandigarh (PGIMER), India **No. of participants:** 5 **Profile of participants:** Members of Civil Society Organizations (CSO), academia, researcher **Duration:** 2 h	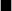 Draft prototype of package components 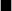 Structure and Layout 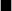 Content 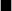 Intended audience and mechanism of delivery	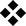 Contents of package: For Health Care Providers (HCPs) -***Booklet*** For patients - ***Disease-specific pamphlets***- ***Short Text Messages*** 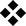 2. Artwork by a professional 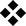 3. Language, translation, metaphors, text 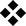 4. Define roles and responsibilities 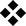 5. Review with revised drafts
**2**^**nd**^ **consultation meeting with CSOs**	**Location:** Department of Community Medicine and School of Public Health, Post Graduate Institute of Medical Education and Research, Chandigarh (PGIMER), India **No. of participants:** 5 **Profile of participants:** Civil Society Organizations, academia, researcher **Duration:** 2 h	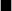 Review of revised drafts of the intervention package 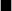 Content & images 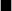 Language	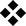 Next plan of action 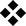 Translation and typing of pamphlets and messages 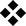 Replace mock pictures with real-time images for booklet and pamphlets 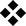 Conduct a workshop with key stakeholders 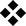 Action plan format
**Workshop with district program managers (NCD & NTCP) and state officials**	**Location:** Advanced Eye Center, Post Graduate Institute of Medical Education and Research, Chandigarh (PGIMER), Chandigarh. **No. of participants: 49 Profile of participants:** State-level and district-level program managers **Duration:**8 h	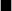 Apprise about the idea 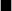 Feedback on booklet 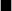 Layout, structure, and content 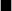 Requirements	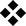 Welcomed the idea of utilizing NCD clinics for cessation & appreciated the contents of the package 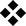 Highlighted higher SLT use in the state 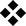 Format for screening all for tobacco use 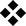 Evolve mechanism/method/framework for rolling out 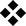 Role of HCPs to be more concise
**State-level consultation meeting**	**Location:** Directorate of Health & Family Welfare, Government of Punjab **No. of participants:** 20 **Profile of participants:** State-level and district-level program managers **Duration:** 4 h	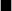 Share the pamphlets and messages developed 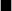 Feedback on content and layout 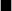 Feedback on content and number of face-to-face counseling sessions 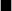 Requirements& Suggestions	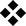 Addition of interaction mechanism in simple language between tobacco and NCD 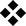 Inclusion of short case stories in the pamphlets 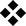 Maximize use of real-time culturally specific images 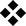 Add locally available resources to the ‘tips to manage craving' section 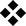 Punjabi- Hindi language revision and typesetting 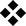 Four customized counseling sessions synced with routine visits tailored to his/her disease, and socioeconomic background after assessing willingness to quit using 5As /5Rs
**After incorporating iterations following an understanding of contextual factors and pre-test** **State-level Advocacy workshop (1 session)**	**Settings:** Directorate of Health and Family Welfare, Punjab **Total participants** = 60 (50 participants from districts + 10 officers & staff from state headquarters) **Duration:** 1 h	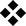 Advocacy of the package with an emphasis on the importance of cessation among NCD patients and utilizing NCD clinics for cessation	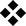 The intervention package received a good response from the participants of the workshop and rendered positive affirmation about its support for the implementation

## Stage 3: Understanding contextual factors of the state and “factor-in” these in the package

The rural population forms 62.51% of the total population in the state of Punjab. The state's economy has predominantly been agrarian and has rich culture and heritage. Punjabi is the state's official language and has a literacy rate of 75.84 %. Punjab has the largest population of Sikhs in India numbering around 16 million comprising 57.69% of the state population. Sikhism is the main religion practiced by about two-thirds of the people (55). Tobacco consumption is strictly prohibited in Sikhism and using tobacco is listed in the Sikh Rehat Maryada- the Sikh code of conduct as one of the four transgressions (Kurahits) (56, 57).

However, the agricultural transformation in Punjab led to internal migration within the population of Punjab from central India. A retrospective review on contextualizing tobacco use in Punjab's social, economic, and political transformation reports that tobacco use in Punjab is determined by the sociopolitical transition from a pre-colonial province to an autonomous Indian State. It also added that although a complex historical process marked these events, the state's retention of its culture offers an interesting aspect of this development. For instance, these events have been marked from the conception of Sikhism to the changing borders of Punjab territory while positioning tobacco use within these boundaries. Additionally, the transitions in the social, economic, and developmental structure of Punjab have played a vital role in the population's health and health behavior. Through various dimensions, the present state of Punjab places the subject of tobacco addiction within the state's social, economic, and political boundaries (58).

Despite tobacco use being a sensitive subject in Punjab, there has been an increase in tobacco use prevalence among adults from 11.7% (Global Adult Tobacco Survey, 2009–2010) (59) to 13.4 % (GATS-2, 2016-2017) (5).

## Cultural adaptation of package to ecological validity model across the eight dimensions

The ecological Validity Model (EVM) has been recurrently applied to the development and adaptation of psychological interventions. The EVM proposes eight dimensions to guide cultural adaptations across- “language, persons, metaphors, content, concepts, goals, methods and context.” The explicit adaptation of interventions across these eight dimensions is thought to increase the ecological and external validity of an intervention. The framework can serve as a guide for developing culturally sensitive treatments and adapting existing psychosocial treatments to specific ethnic minority groups (60). The photographs from the community that were used in the disease-specific pamphlets were real-time and culturally relatable. Couplets and adages from local and religious contexts were incorporated into the intervention package. The text messages and pamphlets were translated into the vernacular language (Punjabi). The local role models were highlighted for adopting a tobacco-free lifestyle. The tips designed to manage craving due to tobacco withdrawal revolved around the available local cultural resources and real-time experiences of tobacco users from the community. Besides, we have tried to adapt various other cultural frameworks as suggested by Reniscow and colleagues (61) and Kreuter (62) and into the development process of the package (Table 3).

**Table 3:** Intervention package: Stages of development and adaptation.

**Stages**	**Dimension (60)**	**Adaptation into the package**	**Strategies (62)**	**Surface/deep structures (61)**
Defining requirements	Context	• The intervention package was developed in consultation with stakeholders that included tobacco from the state, civil society organizations (CSO) functioning in the region, program managers, and health care providers who are providing services in the state's public health system and indigenous to the population served. • This step was taken in order to better understand and incorporate the socio-cultural context into the intervention package. • Multiple consultative meetings and workshops were undertaken during the process. • During the pre-test, in-depth interviews were conducted with each category of HCPs and program managers to identify potential barriers and drivers. • HCPs were oriented on culturally appropriate ways of discussing sensitive topics concerning tobacco use in the state along with patient-centric advice by tailoring advice to their current NCD and background.	Constituent-involving	Surface
Creation of intervention	Language	• The package was created, adapted, and translated into the regional language of “Punjabi”. Also, it was adapted to Hindi as well. The booklet for health care providers was developed in English.	Linguistic	Surface
	Persons	• The text was minimized and emphasis was placed on the pictorial representation of the visuals for a better understanding of poorly literate individuals. • Real-time images were used for better representation. • Short case vignettes were adapted into the disease-specific pamphlets and booklet reflecting the problem and management strategies. • The state attracts a migratory population from central India which is largely Hindi-speaking. Since HCPs working in the system are from the state itself, they are well-versed in the local language and were encouraged to use the language at the convenience of the patient (Hindi/Punjabi).	Peripheral	Surface
	Metaphors	• The material developed had culturally relevant themes and content specific to the group. Common metaphors and couplets from Punjabi literature and religious text were incorporated into the disease-specific pamphlets. • As religion is an important and strong element of the culture of Punjab and we have incorporated excerpts from religious texts into the package.	Socio-cultural	Deep
	Content	• Locally available resources to manage cravings (dried coconut, ginger, fennel seeds, visit *Gurudwara* (religious shrine), and withdrawal are highlighted in the content for the users which was based on the values, belief system, and important aspects of the Punjabi lifestyle. • Based on feedback from tobacco users during the pre-test and activities to be used for managing craving were incorporated.	Socio-cultural	Surface
Delivery of intervention	Concepts	• During training workshops, the concept of confidentiality and privacy were reiterated to the health care providers. • Technical terminology was adapted into local terms to match the literacy level of participants.	Evidential	Surface
	Goals	• Tobacco use is a taboo practice many users do not disclose their tobacco use status due to the fear of being an outcast (socially boycotted). Therefore, they do not seek help to quit on their own. • Hence, we intended to harness the potential of opportunistic cessation support during their routine visit for a consultation.	Socio-cultural	Deep
	Methods	• The existing health care providers within the health care system were trained to deliver cessation intervention during the regular consultation/follow-up visits of the patients to the clinic. • The acceptance of advice offered by healthcare providers among tobacco users also increases because of faith, existing trust, and the relationship that patients have with their treating HCPs. • Appropriate revisions were incorporated into the package based on the findings of the pre-test.	Constituent-involving, Evidential	Surface

## Stage 4: Pre-test of the package

A pre-test of the intervention package was carried out. Each respondent was given a copy of the booklet, disease-specific pamphlets, and short text messages, and feedback was sought on the format, content, and delivery. We conducted five to six in-depth interviews with each category of stakeholder (not involved in the designing process- program managers, medical officers, counselors, nurses, public health experts) and tobacco users. Each interview lasted for 25–30 min. The interview data were analyzed using thematic analysis. The background characteristics of the participants are given in Table 4. The interviews were transcribed followed by data extraction and an analysis worksheet was made. Thereafter, codes were generated and categories were made. Categories were clubbed into themes and sub-themes. The major themes, sub-themes, and codes that emerged from the data are summarized in Table 5.

**Table 4:** Background characteristics of the participants.

	**In-Depth Interview category (*****N*** = **34)**												
**Characteristics**		**Medical officer (*****n*** = **6)**		**Program Officer (*****n*** = **6)**		**Counselor (*****n*** = **6)**		**Nurse (*****n*** = **5)**		**Public Health Experts (*****n*** = **6)**		**Tobacco user (*****n*** = **5)**	
		***n***	**%**	***n***	**%**	***n***	**%**	***n***	**%**	***n***	**%**	***n***	**%**
Gender	Female	2	33.3	3	50	5	83.3	4	80	2	33.3	-	
	Male	4	66.6	3	50	1	16.7	1	20	4	66.6	5	100
Age (years)	Mean ± SD	34.8 ± 2.4		50.7 ± 10.3		32.8 ± 4.9		37.5 ± 5.1		40.5 ± 12.6		44.4 ± 7.3	
	Median (IQR)	34.5 (33.2–37.2)		55.0 (50.0–57.0)		1.0 (28.7–38.5)		39.0 (32.2–41.2)		36.5 (32.0–47.0)		42.0 (39.5–50.5)	
Years of service at the current position (for HCPs only) #Duration of tobacco use (for users only)	Mean ± SD	5.0 ± 1.7		4.1 ± 1.8		4.3 ± 1.9		4.7 ± 3.2		8.5 ± 7.5		#14.6 ± 7.3	
	Median (IQR)	4.5 (3.7–6.5)		5.0 (3.0–6.0)		5.0 (2.5–6.0)		4.5 (2.0–7.7)		4.5 (3.0–17.0)		#17.0 (7.0–21.0)	

**Table 5:** Themes and codes as per thematic analysis.

**Theme**	**Sub-themes**	**Codes**
1. Strengthening of certain factors	1.1 Individual level 1.2 Structural level	• Specific training on tobacco cessation • Provision of adequate IEC material • Supportive supervision • Political and administrative commitment • Performance-based incentives • Administrative coordination • Filling up of staff deficiency • Building an institutional framework
2. Potential enablers	2.1 Healthcare providers 2.2 Tobacco users 2.3 Healthcare facilities	• HCPs are advocates and role models • Good rapport • Synchronized sessions with routine visits • Services under one roof • Client-centered therapy • Utilization of existing HR & resources • User-friendly & vernacular language • Increased outreach of cessation services
3. Potential challenges	3.1 Healthcare providers 3.2 Tobacco users 3.3 Healthcare facilities	• Lack of trained & motivated manpower • Inertia from HCPs and workload on staff • Lack of health literacy • Patients not reporting for follow-ups • Avoiding peers who are users and when ideal • Lack of marketing of such a program • High footfall
4. Suggestions for improvisation	• Field testing by a pilot at the district level • Contents of package	• Pilot of the package & provision of additional resources • Inter & Intra administrative coordination • A sensitive issue, understanding the social fabric, creating a common HTN+DM IEC • The intervention package is open to revision and more pictorial representation, modify it acc. to patient-specific population • Tips: drinking water, playing with pets, and children, sports • Sharing success stories/case stories and adding a contact number • Involvement of family, and children, highlight social perks • Disease, mobile messages, money wastage, case stories

These suggestions were incorporated into the existing package and revisions were made.

## Stage 5: Micro detailing and expansion of the package by drawing on existing theories of the cascade model and TTM model

**Figure 2:**
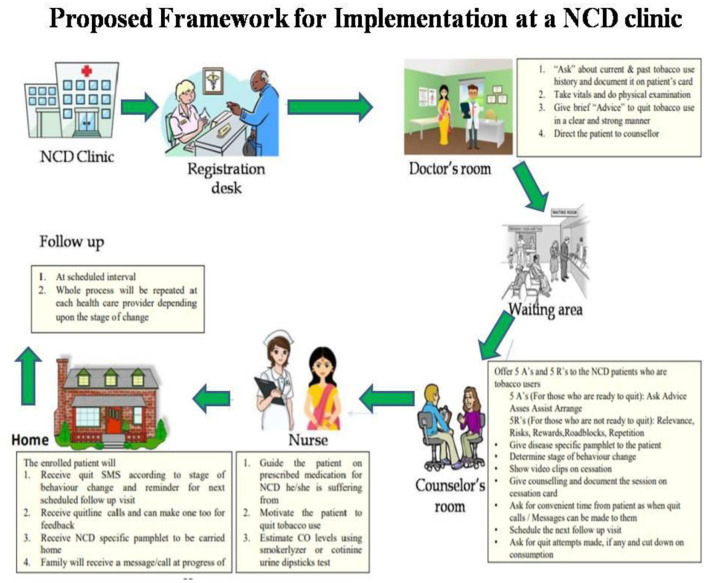
Proposed implementation framework at NCD clinics.

**“Cascade training”** is extensively used as an effective and efficient approach for addressing the scarcity of healthcare professionals in LMICs by upgrading their skills and eventually improving their job performance and participation (63). The objective remains to enhance the diffusion of innovation. The cascade model is used mainly for in-service training, as this strategy can train large numbers of people within a limited time (64). A major finding from the in-depth interviews was that HCPs and program managers highlighted the need for a specific capacity-building training program on tobacco cessation. Therefore, using this as an opportunity, we trained the in-service human resource and associated program managers at the state and district-level training workshops. Based on the feedback, training objectives were set and supported with copies of intervention package components. Besides, the master trainers were subject experts with a comprehensive understanding of the knowledge and skills required to be transferred. In addition, the role and function of each stakeholder were defined in the proposed framework (Figure 2). A systematic review of TTT in health and social care found that the TTT programs helped to increase knowledge, improve clinical behavior, and produce better patient outcomes (65).

## Transtheoretical model

**The Transtheoretical Model (TTM)** of behavior change provides a framework for both understanding and measuring behavior change. It facilitates the development of individually tailored intervention strategies that can be easily modified to fit diverse populations (66). TTM recognizes change as a process toward desired behavior through a series of stages. While progression through the Stages of Change can occur linearly, a nonlinear progression is common. It suggests that smokers move through a series of motivational stages before they manage to stop smoking. These are *pre-contemplation* (no thoughts of quitting), *contemplation* (thinking about quitting), *preparation* (planning to quit in the times to come, say days or weeks), *action* (quitting successfully for up to for a considerable duration, usually, six months), and *maintenance* (no smoking for more than 6 months). According to this TTM, programs that help people to stop smoking should be matched to their stage of readiness to quit (67). The backdrop of this model was utilized in developing and sending stage-based short text messages to tobacco users. The assessment was carried out using the “stage of change” questionnaire. Besides, these were bilingual (Punjabi and Hindi) suited according to the preference of the patient.

## Stage 6: Evolving analysis plan

The developed intervention package would be implemented at two district-level NCD clinics through a randomized controlled trial (RCT) comparing two groups (one receiving package and the other receiving usual care). These participants would be followed up for 1 year with follow-ups at the 3^rd^ month, 6^th^ month, 9^th^ month, and 12^th^ month. Besides, the biochemical assessment would also be undertaken for the participants to assess their quit status using plasma cotinine levels (LC-MS). However, this would be restricted only to two pilot districts. In addition, a mixed-methods study would also be undertaken to understand the barriers and facilitators of package implementation from the perspective of healthcare providers and program managers. The emphasis on the qualitative part would assist to understand the factors in a detailed manner. Also, an economic evaluation would be carried out for the package development phase and implementation phase using a health systems perspective. Cost analysis of the intervention package is important from an implementation viewpoint, for policymakers to help resource allocation between various interventions available in the basket and sustain newly developed programs.

## Discussion

**Figure 3:**
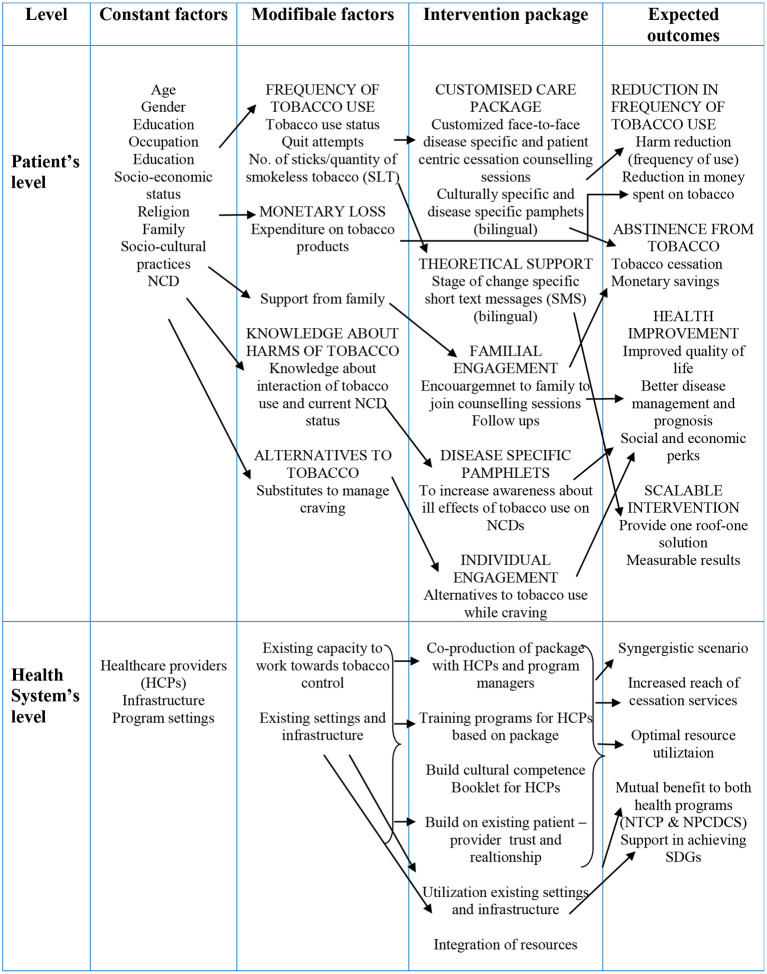
Conceptual framework.

During this exercise, we developed a multi-component culturally-specific, disease-specific, and patient-centric tobacco cessation intervention package utilizing multiple approaches of intervention development for the health care providers and patients attending NCD clinics in Punjab, India. We adapted and incorporated successful non-pharmacological strategies from existing cessation interventions available. The novelty of the intervention developed lies in it being culturally tailored, patient-centric, NCD specific, bilingual (Punjabi/Hindi)- SMS, pamphlets and counseling sessions, and encouragement to family members to be involved in counseling sessions. While developing and implementing an intervention, consideration of tobacco users' socio-cultural and patient-centric approach makes it a robust strategy that is adaptable in similar settings elsewhere. Further, it better assists HCPs who can then enhance their potential to deliver the required service by easy understanding of the patient's cultural aspects through the medium of intervention. The intervention underlines both the negative implications of tobacco and the benefits of quitting, as well as how quitting is relevant to the patient's health, social, and family contexts. The entire package was developed in co-production with relevant stakeholders through a series of exhaustive consultative meetings and workshops. It aims to provide “opportunistic quitting assistance” to NCD patients who are currently using tobacco in any form (smoked /smokeless tobacco). In addition, the package enhances the capacity development of present human capital at NCD clinics via training workshops and helps to build cultural competence. This has the potential to optimize resources and long-term sustainability within the existing healthcare system. The use of mobile technology to deliver culturally, linguistically, and stage of change appropriate short text messages could be an efficient strategy to trigger constant motivational behavior change at regular intervals, to compensate for the loss of motivation over a specific duration, which is commonly observed when it comes to sustaining the attitude and practice of changed behavior. There is inadequate focus on the healthcare promotion aspects concerning NCD risk factors in the present educational landscape of medical education in India (68). Besides, during the pre-test, we gauzed feedback from users as well as beneficiaries that helped to improvise the package. The conceptual framework of the intervention package is represented in Figure 3.

The study's limitation includes a purposive selection of the participants for the IDI therefore the views and suggestions may not be generalizable. The cascade model is often criticized for distortion of the messages transferred during the training, resulting in dilution through miscommunication and different interpretations of the same messages (64). In addition, the TTM model ignores the social context in which change occurs (such as SES and income). The model assumes that individuals make coherent and logical plans in their decision-making process when this is not always true. Given that tobacco use is a sensitive issue in the intervention implementation state, we expect that culture-specific intervention materials would bridge the gap. Furthermore, the results may help healthcare decision-makers introduce large-scale projects to provide culturally relevant patient-centric care and improve care services for diversified socio-cultural communities.

## Data availability statement

The data supporting the study findings are available upon request from the corresponding author (SGo). The data are not available publicly due to restrictions (such as the presence of information that may compromise the confidentiality of research participants).

## Ethics statement

The Ethics approval was granted by the Institute Ethics Committee (IEC) of the Post Graduate Institute of Medical Education and Research (PGIMER), Chandigarh, India (IEC number: INT/IEC/2017/1361). Prior permissions were obtained from the State Tobacco Control Cell and the NCD Control Cell, Department of Health and Family Welfare, Government of Punjab, India. The main study's protocol has been registered with India's Clinical Trials Registry, with the registration number CTRI/2018/01/011643. The participants provided their written informed consent to participate in this study. Written informed consent was obtained from the individual(s) for the publication of any potentially identifiable images or data included in this article.

## Author contributions

SGo conceptualized the idea. SGo, BM, and SGr developed the methodology. GB performed the data collection, analysis, and prepared the first draft. GS and SGi facilitated the administration. LS, RS, SGo, BM, SGr, GS, and SGi gave technical inputs to the first draft. The final draft was approved by all authors. All authors contributed to the article and approved the submitted version.

## Funding

This current manuscript is the Ph.D. work of GB. She is a recipient of the Junior Research Fellowship by the Indian Council of Medical Research [ICMR-JRF (No. 3/1/3/JRF-2016/HRD)] to pursue her Ph.D. Program.

## Conflict of interest

The authors declare that the research was conducted in the absence of any commercial or financial relationships that could be construed as a potential conflict of interest.

## Publisher's note

All claims expressed in this article are solely those of the authors and do not necessarily represent those of their affiliated organizations, or those of the publisher, the editors and the reviewers. Any product that may be evaluated in this article, or claim that may be made by its manufacturer, is not guaranteed or endorsed by the publisher.
